# Epithelial-myoepithelial carcinoma of the lung: a case report

**DOI:** 10.1186/s40792-018-0482-8

**Published:** 2018-07-09

**Authors:** Yasuhiro Nakashima, Riichiro Morita, Akiko Ui, Kuniko Iihara, Takuya Yazawa

**Affiliations:** 1 Department of Chest Surgery, Tokyo Yamate Medical Center, 3-22-1 Hyakunin-cho, Shinjuku-ku, Tokyo, 169-0073 Japan; 2 Department of Pathology, Tokyo Yamate Medical Center, 3-22-1 Hyakunin-cho, Shinjuku-ku, Tokyo, 169-0073 Japan; 3 Department of Thoracic Surgery, Tokyo Medical and Dental University, 1-5-45, Yushima, Bunkyo-ku, Tokyo, 113-8519 Japan; 4 Department of Pathology, Dokkyo Medical University, 880 Kitakobayashi, Mibu-machi, Shimotsuga-gun, Tochigi, 321-0293 Japan

**Keywords:** Pulmonary epithelial-myoepithelial carcinoma (P-EMC), Epithelial-myoepithelial tumor, Pulmonary salivary gland-type tumor, Lung cancer

## Abstract

**Background:**

Pulmonary epithelial-myoepithelial carcinoma (P-EMC) is a rare subset of salivary gland-type tumors of the lung. Because of its rarity and unproven malignant potential, the optimal therapy for P-EMC has not been defined. Here, we report a typical case of P-EMC and a review of the literature to consider appropriate treatment.

**Case presentation:**

A 54-year-old woman presented with an abnormal lung shadow on a routine chest X-ray. A chest computed tomography (CT) scan verified an 18-mm endobronchial nodule on the middle lobe. We performed a bronchoscopic biopsy, and the patient was diagnosed with P-EMC. After confirming the absence of tumors in the salivary glands, she underwent a right middle lobectomy along with hilar and mediastinal lymph node dissections. Currently, the patient is doing well, without any sign of recurrence 3 years after surgery.

**Conclusions:**

Although a majority of P-EMC cases, as in our case, behave indolently, several poor progression cases have been reported. For distinguishing the minor malignancy cases from others, histological findings such as myoepithelial anaplasia could be a predictive factor. Complete resection is needed to evaluate the whole tumor, because P-EMCs often show histological heterogeneity. Moreover, incomplete excision may be a poor prognostic factor. Although lobectomies as well as lymph node dissections, sleeve lobectomies, or pneumonectomies are routinely performed for complete resection, further investigation is required to establish the optimal treatment strategy.

## Background

Pulmonary epithelial-myoepithelial carcinoma (P-EMC) is a rare subset of salivary gland-type tumors of the lung. Although it is generally regarded as a low-grade malignant tumor and typically behaves indolently [1], distant metastases and recurrences occasionally occur. Some pathologists describe the malignant potential of P-EMC as “unproven,” rather than “low-grade malignant” [2]. Because of its rarity and unproven malignant potential, optimal therapy for P-EMC has not been defined. Here we report a typical case of P-EMC and a review of the literature to consider appropriate treatment.

## Case presentation

**Fig. 1:**
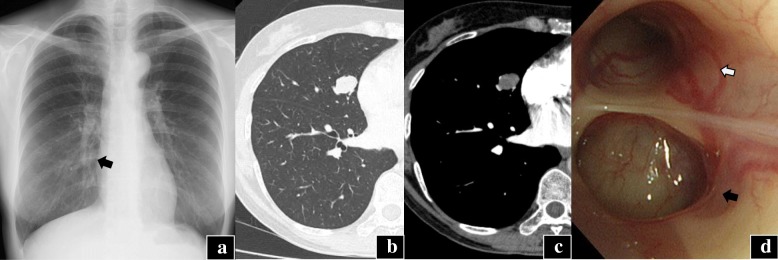
Medical imaging findings of the nodule. **a** Chest X-ray reveals a 2-cm shadow in the right middle lung field (black arrow). **b**, **c** CT scan reveals an 18-mm lobulated nodule. **d** Bronchoscopy shows the endobronchial nodule reducing the lumen of right B^5^a sub-segmental bronchus (black arrow) and the remaining patency of the B^5^b sub-segmental bronchus (white arrow)

A 54-year-old female patient presented with an abnormal shadow discovered on a routine chest X-ray. She had a history of smoking 4–5 cigarettes per month for 5 years but quit over 10 years ago. Her past medical history included a colorectal benign polyp resected by endoscopy. She did not have respiratory symptoms and laboratory findings were unremarkable. The serum levels of the tumor markers (carcinoembryonic antigen, squamous cell carcinoma antigen, and cytokeratin 19 fragment) were within normal limits. A chest radiograph showed a nodular shadow at the right middle lung field (Fig. 1a), and a computed tomography (CT) scan confirmed an 18-mm lobulated nodule at the middle lobe (Fig. 1b, c). An F^18^-fluoro-deoxy-glucose positron emission tomography/CT (FDG-PET/CT) scan did not indicate abnormal FDG uptake. Bronchoscopy showed the round, tan, solid endobronchial nodule reducing the lumen of the right subsegmental bronchus (B^5^_a_) (Fig. 1d). A bronchoscopic biopsy was performed, and the patient was diagnosed with an epithelial-myoepithelial carcinoma (EMC). Examination of otolaryngologist and magnetic resonance imaging (MRI) of the head revealed no salivary gland pathologies. A right pulmonary middle lobectomy was performed, along with hilar and mediastinal lymph node dissections.

**Fig. 2:**
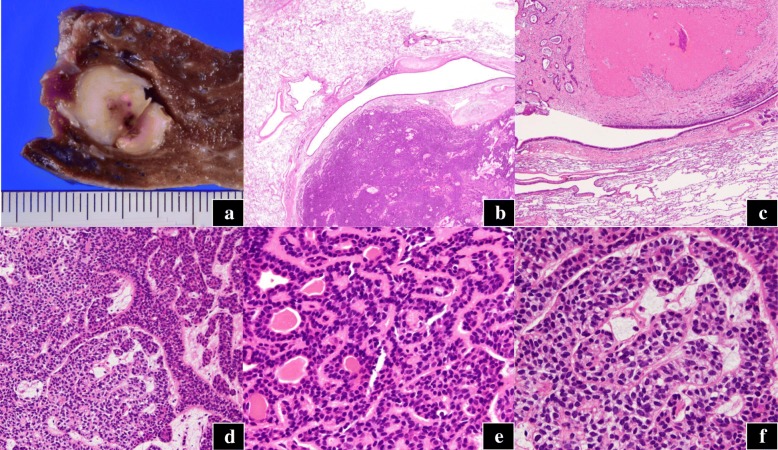
Macroscopic and microscopic findings of the pulmonary nodule. **a** Cut surface of the surgical specimen. A white, well-circumscribed tumor with lobulated appearance is found along the bronchial wall. Microscopic findings (hematoxylin and eosin) reveals **b** a submucosal tumor oppressed by the adjacent bronchioles and **c** a part of the tumor is necrotic (original magnification; **b** ×20; **c** ×40). **d** Two different components are observed: **e** the duct-forming component and **f** outer multilayered polygonal cells with clear cytoplasm (original magnification; **d** ×200; **e**, **f** ×400)

**Fig. 3:**
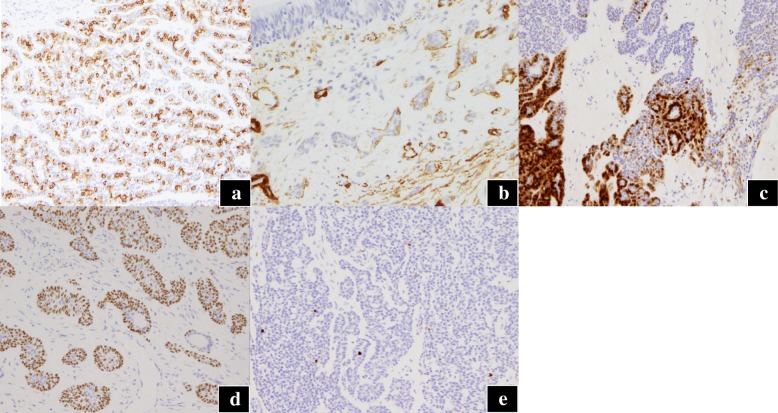
Immunohistochemical staining of the tumor. **a** Immunoreactivity in epithelial cells for cytokeratin 7; immunoreactivity in myoepithelial cells for **b** smooth muscle actin (SMA), **c** S100, and **d** p63. **e** The Ki-67 labeling index was < 5% (original magnification; **a**, **c**, **e** ×200; **b**, **d** ×400)

The tumor was measuring 15 mm in diameter and had a white surface; it was well-circumscribed and was present along the bronchial wall (Fig. 2a). On histological examinations, the tumor was located in the submucosal layer of the bronchus, oppressing the adjacent bronchioles, and partly necrotic (Fig. 2b, c). The tumor consisted of two different components: the duct-forming epithelial cells and outer multilayered polygonal cells with clear cytoplasm (Fig. 2d–f). Duct-forming epithelial cells were positive for cytokeratin 7, while the outer cells were negative (Fig. 3a). The outer cells were positive for S100 protein, smooth muscle actin (SMA), p63, and cytokeratin 5/6 (Fig. 3b–d), suggesting myoepithelial phenotype. Neither vascular, lymphatic, nor neural invasion was observed, and the mitotic rate is rare. The Ki-67 labeling index was less than 5% (Fig. 3e). No mutation was found in the *KRAS* or *EGFR* gene. We finally diagnosed the patient with P-EMC. The patient is doing well without any sign of recurrence 3 years after surgery.

## Discussion

P-EMC is a tumor characterized by biphasic morphology, consisting of an inner layer of duct-like structures made of epithelial cells and a surrounding layer of myoepithelial cells immunoreactive for S-100 and smooth muscle actin [1]. Precise diagnosis of this tumor via preoperative bronchoscopy is difficult because of its revealing heterogeneity; however, biphasic features in our biopsy specimen allowed us to make the diagnosis of P-EMC before surgery.

Even when a preoperative diagnosis of P-EMC is possible, optimal treatment methods and follow-up periods have not been established due to the tumor’s unproven malignant potential [2]. Our review of the literature revealed a total of 56 P-EMC cases, including our case, in the English literature (Tables 1 and 2) [2–27]. We found reported cases of 32 females and 24 males, with an average age of 56 years (age range, 7–81 years). Forty-five cases had tumors localized in the central airway within segmental bronchi and appeared to be endobronchial masses. On the contrary, 11 cases had tumors localized in the pulmonary parenchyma [3, 14, 18, 23, 27], and 5 of these cases were reported with tumors clearly presenting as intraparenchymatous masses without apparent connection with a bronchus [3, 18, 27]. Because endobronchial localizations and the histologic features mimic those of salivary gland tumors, P-EMCs are regarded as originating from the epithelium of submucosal bronchial glands. However, the existence of these tumors in the peripheral lung tissue suggests that P-EMC might originate from primitive cells [3]. In the 25 cases we reviewed, patients presented with symptoms of bronchial obstruction such as productive cough, fever, and dyspnea. Although our patient was asymptomatic, obstruction of her sub-segmental bronchus would have eventually caused symptoms. As one of the reasons why poor clinical courses in cases of P-EMC are fewer than those of salivary gland EMC, it is considered that obstructive bronchial symptoms often appear [26]. In many cases we reviewed, as in our case, CTs demonstrated that the masses had comparatively clear boundaries and homogeneous densities. While the most frequently reported P-EMCs do not reveal abnormal FDG uptake in FDG-PET/CT scans as in this case, three cases revealed active FDG uptake [20, 23, 25], and one of those had hilar and subcarinal lymph node metastasis [25].

**Table 1:** Review of P-EMC cases: Clinical characteristics and surgical procedure

Year	Author	Age	Sex	Obstructive airway symptoms	Location	Location (endo-bronchial or not)	Surgical procedure	Mediastinal lymph node dissection	Size (cm)
1994	Moran CA et al. [3]	1: 47	F	+	LMB	E	Pneumo	NA	2.5
		2: 45	F	−	LLL	P, U	L	NA	2.5
		3: 42	F	−	RLL	E	L	NA	2.5
		4: 57	M	−	RUL	P, U	L	NA	2.0
		5: 58	F	+	LUL	E	L	NA	2.0
		6: 35	F	+	RLL	E	L	NA	16.0
		7: 67	M	+	RUL	E	L	NA	6.0
		8: 69	F	−	LLL	P, U	L	NA	2.0
1994	Nistal et al. [4]	55	F	+	RULB	E	L	NA	2.0
1995	Tsuji et al. [5]	66	M	NA	RMB	E	Pneumo	NA	16
1997	Wilson RW et al. [6]	55	F	+	LLSB	E	L	NA	3.9
1998	Shanks et al. [7]	67	M	+	LLLB	E	L	NA	1.3
1998	Ryska et al. [8]	47	F	+	RULB	E	B	–	NA
2001	Fulford LG et al. [9]	1: 55	F	+	RMB	E	Pneumo	NA	5.0
		3: 56	M	+	Lobe bronchus side unstated	E	L	NA	NA
		4: 57	F	+	LMB	E	Pneumo	NA	1.5
		5: 54	F	NA	RULB	E	L	NA	1.5
2001	Pelosi et al. [2]	47	M		LULB	E	SL	+	1.5
2003	Doganay et al. [10]	73	M	+	LLLB	E	Pneumo	+	5
2004	Ru et al. [11]	73	M	+	LULB	E	L	−	3.8
2007	Chao et al. [12]	43	F	+	LMB	E	B	−	NA
2007	Musulimani et al. [13]	74	M	+	LMB	E	B	−	NA
2007	Chang et al. [14]	1: 54	F	−	RLL	P, *1	W	−	2.6
		2: 62	F	−	LLL	P, *1	W	−	2
		3: 58	F	+	RML	P, *1	W	−	1.2
		4: 57	F	−	LUL	P, *1	W	−	0.8
		5: 52	F	−	RUL(bilateral nodules)	P, *1	W	−	1.2
2009	Nguen et al. [15]	1: 38	M	−	LLL	E	L	−	5
		2: 48	M	−	RUL	E	L	−	2.5
		3: 52	F	−	LLL	E	L	−	3
		4: 54	M	−	RUL	E	L	−	3
		5: 56	F	−	LMB	E	Pneumo	NA	4.2
2009	Rosenfeld et al. [16]	7	M	−	RLSB	E	L	N/A	3.6
2011	Nishihara et al. [17]	81	M	−	RULB	E	Biopsy only, BSC	−	NA
2011	Munoz et al. [18]	76	F	−	RUL	P, U	L	NA	2.7
2011	Kang et al. [19]	1–2: median 57.0	M(1) F(1)	NA	LUL(1) LLL(1)	NA	SL(1) Pneumo(1)	+(2)	Median 6.9
2012	Arif et al. [20]	57	M	–	Rt. Intermedius bronchus	E	Bi-L	NA	1.2
2013	Zhu et al. [21]	1~ 7: median 63 (36–75)	M(3) F(4)	+(3)	RMB(2) RUL(1) RLL(2) LUL(1) LLL(1)	NA	L(5) SL(1) Pneumo(1)	NA	Median 2.5 (1.3–4.0)
2013	Konoglou et al. [22]	34	M	+	Trachea	E	Resection of five tracheal rings	−	1.15
2014	Cho et al. [23]	51	F	−	LUL	P, NA	L	+	3.3
2014	Song et al. [24]	1: 52	F	+	LLL	E	L	NA	12
		2: 66	M	+	LUL	E	SL	NA	1.8
		3: 60	M	−	LUL	E	L	NA	0.7
		4: 61	M	+	RUL	E	L	NA	1.5
		5: 63	F	+	Trachea	E	B	−	2
2015	Cha et al. [25]	53	F	+	Rt. intermedius bronchus	E	Bi-L(VATS)	+	2.2
2015	Tajima et al. [26]	72	F	−	LBSB	E	L(VATS)	+	3.8
2016	Shen et al. [27]	58	M	−	LLL	P, U	NA(VATS)	NA	1.3
Current case		54	F	−	RMLB	E	L	+	1.5

*M* male (number of people), *F* female (number of people), *N/A* not available, *LMB* left main bronchus, *LLL* left lower lobe, *RLL* right lower lobe, *RUL* right upper lobe, *LUL* left upper lobe, *RULB* right upper lobe bronchus, *RMB* right main bronchus, *LBSB* left basal segment bronchus, *LLLB* left lower lobe bronchus, *LULB* left upper lobe bronchus, *RML* right middle lobe, *RLSB* right lower lobe segment bronchus, *L()* lobe(number of people), *Rt.* right, *RMLB* right middle lobe bronchus, *E* endobronchial, *P* pulmonary parenchyma, *U* unrelated to a bronchus, *1 located in the periphery of the lung, did not involve any large bronchi, close proximity to a small caliber airway, *Pneumo* pneumonectomy, *L* lobectomy, *B* endobronchial excision, *SL* sleeve lobectomy, *W* wedge resection, *BSC* best supportive care, *Bi-L* bi-lobectomy, *VATS* video-assisted thoracic surgery

**Table 2:** Review of P-EMC cases: Cases and clinicopathological features

Year	Author	Predominant component	High mitotic rate/necrosis/Ly,V,N invasion	Ki-67	p53	Metastasis	F/U (months)
1994	Moran CA et al. [3]	G	−/−/−	NA	NA	Free	FOD(72)
		M	(2–3/10HPF)/+/−	NA	NA	Free	FOD(48)
		M	−/−/−	NA	NA	Free	NA
		M	(2–3/10HPF)/+/−	NA	NA	Free	Died of surgery(0)
		G	−/−/−	NA	NA	Free	NA
		M	(5–10/10HPF)/+/−	NA	NA	Free	Recurred LN mets after 2 years
		M	(5–10/10HPF)/+/+	NA	NA	Free	Recurred after 3 years in trachea After CRTx, mets to multiple organs Died of P-EMC.
		M	(2–3/10HPF)/−/−	NA	NA	Free	NA
1994	Nistal et al. [4]	G	Scanty/NA/−	NA	NA	Free	FOD(24)
1995	Tsuji et al. [5]	M	Rarely/+/−	NA	NA	Free	FOD(36), died of unrelated disease.
1997	Wilson RW et al. [6]	G	−/−/−	NA	NA	Free	FOD(7)
1998	Shanks et al. [7]	G	(1/20HPF)/−/−	NA	NA	Free	NA
1998	Ryska et al. [8]	G	NA/NA/NA	NA	NA	Free	FOD(13)
2001	Fulford LG et al. [9]	G	(1/20HPF)/−/−	2–10%	NA	Free	FOD(8)
		G	(1/20HPF)/−/−	1–2%	NA	Free	FOD(60)
		M	−/−/−	< 1%	NA	Free	FOD(96)
		M	(1/20HPF)/+/−	1–2%	NA	Free	FOD(84)
2001	Pelosi et al. [2]	NA	−/−/−	G)1.5%, M)12%	−	Free	FOD(6)
2003	Doganay et al. [10]	NA	Few/+/−	G)1%, M)8%	−	Free	FOD(34)
2004	Ru et al. [11]	G	A few/NA/−	< 5~20%	+	Free	FOD(8)
2007	Chao et al. [12]	NA	−/NA/NA	2.8	+	Free	FOD(6)
2007	Musulimani et al. [13]	NA	−/NA/NA	NA	NA	Free	recurred bilateral lung lesions, Tumor bearing(48)
2007	Chang et al. [14]	G	Rare/NA/NA	< 5%	NA	NA	FOD(31)
		G	Rare/NA/NA	< 5%	NA	NA	FOD(14)
		G	Rare/NA/NA	< 5%	NA	NA	FOD(13)
		G	Rare/NA/NA	< 5%	NA	NA	FOD(78)
		G	Rare/NA/NA	< 5%	NA	Bilateral lung nodules.	No recurrence(5) not changed in appearance on a follow-up CT
2009	Nguen et al. [15]	NA	Rare/NA/ 1 case: Ly(+), V(+), N(+)	NA	NA	1/5 case: infiltrated peribronchial tissue and LN metastasis.	FOD(4)
		NA		NA	NA		FOD(12)
		NA		NA	NA		NA
		NA		NA	NA		FOD(12)
		NA		NA	NA		FOD(4)
2009	Rosenfeld et al. [16]	NA	Rare~few/−/NA	NA	NA	The biphasic neoplastic cells replaced part of a lymph node.	FOD(12)
2011	Nishihara et al. [17]	NA	(−/−/NA)(biopsy)	(10%)(biopsy)	NA	NA/skull metastasis	NA
2011	Munoz et al. [18]	G	−/−/−	NA	NA	Free	NA
2011	Kang et al. [19]	NA	NA	NA	NA	Free	1/2 case: recurrence; ipsilateral lung, pneumonectomy
2012	Arif et al. [20]	G	(2–3/10HPF)/−/NA	2–3%	NA	Free	FOD(9)
2013	Zhu et al. [21]	NA	NA/NA/NA	NA	NA	Free	5-year OS, 100%, 1 case: mets to bone within 3 years, Others: FOD(~60)
2013	Konoglou et al. [22]	NA	NA/NA/NA	Particularly low	NA	−	FOD(24)
2014	Cho et al. [23]	NA	A few/NA/NA	NA	NA	Free	FOD(16)
2014	Song et al. [24]	M(> 95%)	−/−/NA	NA	NA	Free	Recurrence(33), Complete pneumonectomy, mets to chest wall(37), Died of P-EMC(117)
		M(30%)	−/+/NA	NA	NA	Free	FOD(75)
		M(60%)	−/−/NA	NA	NA	Free	FOD(33)
		M(70%)	−/−/NA	NA	NA	Free	FOD(1)
		M(40%)	−/−/NA	NA	NA	Free	FOD(10)
2015	Cha et al. [25]	M	NA/+/−	G) < 1% M) 40%	NA	Hilar LN+subcarinal LN+	Adj Chemo
2015	Tajima et al. [26]	M(70–90%)	A few/−/V(+)	G)1.6%, M)2.8–14.2%	a few +	Free	FOD(4)
2016	Shen et al. [27]	NA	NA/NA/NA	NA	NA	Free	FOD(8)
Current case		G	Rare/+/−	< 5%	NA	Free	FOD(36)

*G* dual layered glands, *M* solid or sheets of myoepithelial cells, *NA* not available, *M()* percentage of the myoepithelial component, *Ly* lymphatic, *V* vascular, *N* neural, *HPF* high-power field, *LN* lymph node, *F/U* Follow-up, *FOD* free of disease, *mets* metastasis, *CRTx* chemotherapy and radiotherapy, *Adj* Adjuvant

Although P-EMC cases are typically indolent, they are potentially malignant, and recurrence and metastasis may occur. Clinical follow-up information is provided for 50 cases in this review. Six cases of recurrence and four cases of metastasis have been reported thus far, and two of the six patients with recurrence died of P-EMC. The size of the tumors varied, ranging from 0.7 to 16 cm in diameter, with an average of 2.5 cm. The size of P-EMC that occurred in the metastasis or recurrence tended to be larger than the average size of P-EMC. The size of P-EMCs causing lymph node metastases or recurrence were 3.6 cm [16] and 2.2 cm [25] or 16 cm [3], 6 cm [3], and 12 cm [24], respectively. All 11 tumors localized in pulmonary parenchyma showed no evidence of recurrence or metastasis.

There are three histological distinct subtypes of P-EMC: one presents with a dual ductal component, which is a defined characteristic feature of this tumor (19 cases including our case); one presents with a solid component mainly consisting of spindle and polygonal-shaped myoepithelial cells (14 cases); and one mainly consists of myoepithelial cells with increased nuclear atypia, called myoepithelial anaplasia (four cases) [3, 25, 26]. For distinguishing the minor malignant cases from others, many pathologists have attempted to identify a specific histopathological finding as a predictive factor. Poor prognostic factors of the salivary gland EMC are often applied to P-EMCs. Seethala et al. reported that positive margin status, presence of angiolymphatic invasion, necrosis, and myoepithelial anaplasia in the EMC in salivary glands were predictors of decreased disease-free survival (DFS). Histology of both patients who died of P-EMC showed myoepithelial cell-predominant features with anaplasia [3, 24]. The other three cases having a component of myoepithelial anaplasia showed the tumor progression: a case recurred 2 years after lobectomy [3], lymph node metastasis was found at the surgery in a case [25], and pulmonary infiltration was found in a case [26]. Therefore, myoepithelial anaplasia could be one of the predictive poor prognostic factors of P-EMC.

Complete resection is needed to evaluate the whole tumor, which usually shows histological heterogeneity. Moreover, incomplete excision may be a predictor of poor prognosis for P-EMC, as it is in salivary gland EMC [28]. Despite the fact that most of P-EMCs are indolent, various kinds of surgical procedures have been frequently performed until now for complete resections. Among the cases we reviewed, the following procedures were performed: a partial resection of the trachea (1 case), lobectomy (28 cases), sleeve lobectomy (4 cases), bi-lobectomy (2 cases), and pneumonectomy (8 cases). Other less-invasive procedures were performed in a few cases—wide edge resection (5 cases), excision by bronchoscopy (4 cases), and biopsy by bronchoscopy (1 case). Chao et al. performed bronchoscopic excision, because the patient refused a surgical procedure and the tumor growth was limited into the bronchial cartilage layer. The doctors argued that curative electrosurgery was an option for management of this low-grade malignancy [12]. In contrast, the case of Musulimani et al. revealed residual and/or recurrent P-EMC 8 months after their patient underwent a bronchoscopy that revealed a bilateral lung metastatic lesion; however, he remained asymptomatic and clinically healthy after 4 years [13]. Therefore, bronchoscopic resection could be a viable option, especially when passive treatment is desired. We think that it is necessary to explain sufficiently to the patient that additional surgical resection is needed in order to examine whether the residual tumor contains elements suggesting poor prognosis.

Among 56 cases we reviewed, 3 cases of metastatic lymph nodes were found at surgery. Moreover, only 7 reported the performance of systematic lymph node dissection. Although the necessity of lymph node dissection is unclear, sampling of lymph nodes to establish the cancer stage is considered a beneficial option, especially if there are any findings that suggest tumor aggressiveness. Therefore, we suggest that evaluation of lymph node metastasis provides valuable information in post-operative follow-up due to the unproven malignant potential of P-EMCs. In salivary gland EMCs, it has been reported that there are long intervals between original treatment and recurrence (mean, 5 years) or metastasis (mean, 15 years) [26]. In our review, there are six recurrent cases after the surgical treatment, and the interval was 8 months (1 case), 2 years (1 case), 3 years (3 cases), or data not available (1 case) [3, 13, 19, 21, 24]. These data indicate that a thorough follow-up of at least 3 years is necessary after surgery.

## Conclusions

Here, we report a case of P-EMC for the rarity. Although the majority of P-EMCs behave indolently as seen in our case, our review indicates that several P-EMCs progress. Histological findings such as myoepithelial anaplasia could be a predictive factor for distinguishing the minor malignant cases from others. Complete resection is needed to evaluate the whole tumor, since P-EMC usually shows histological heterogeneity and since incomplete excision may be a poor prognostic factor. Until now, lobectomies, as well as lymph node dissections, sleeve lobectomies, or pneumonectomies, have been frequently performed for complete resection of P-EMC. Further investigation is required to establish the optimal treatment strategy.

## Abbreviations

CT: Computed tomography
DFS: Disease-free survival
EMC: Epithelial-myoepithelial carcinoma
FDG-PET: F^18^-fluoro-deoxy-glucose positron emission tomography
HGT: High-grade transformation
MRI: Magnetic resonance imaging
P-EMC: Pulmonary epithelial-myoepithelial carcinoma
SMA: Smooth muscle actin
